# Supplementary material for: Diverging asymmetry of intrinsic functional organization in autism
Source: Mol Psychiatry. 2023 Aug 16;28(10):4331–41. doi: 10.1038/s41380-023-02220-x (PMC10827663; doi:10.1038/s41380-023-02220-x)
Supplement: Supplementary file 1 — Supplementary Results [file 41380_2023_2220_MOESM1_ESM.pdf]

Supplementary Results for
"Diverging asymmetry of intrinsic functional organization in autism"

Bin Wan [1,2,3,4 *], Seok-Jun Hong [5], Richard A.I. Bethlehem [6], Dorothea L. Floris [7,8], Boris C. Bernhardt [9], Sofie L. Valk [1,4,10 *]

1. *Otto Hahn Research Group Cognitive Neurogenetics, Max Planck Institute for Human Cognitive and Brain Sciences, Leipzig, Germany.*
2. *International Max Planck Research School on Neuroscience of Communication: Function, Structure, and Plasticity (IMPRS NeuroCom), Leipzig, Germany.*
3. *Department of Cognitive Neurology, University Hospital Leipzig and Faculty of Medicine, University of Leipzig, Leipzig, Germany.*
4. *Institute of Neuroscience and Medicine (INM-7: Brain and Behaviour), Research Centre Jülich, Jülich, Germany.*
5. *Centre for Neuroscience Imaging Research, Institute for Basic Science, Department of Global Biomedical Engineering, Sungkyunkwan University, Suwon, South Korea.*
6. *Department of Psychology, University of Cambridge, Cambridge, UK.*
7. *Department of Psychology, University of Zürich, Zürich, Switzerland.*
8. *Department of Cognitive Neuroscience, Donders Institute for Brain, Cognition and Behaviour, Radboud University Nijmegen Medical Centre, Nijmegen, Netherlands.*
9. *McConnell Brain Imaging Centre, Montréal Neurological Institute and Hospital, McGill University, Montréal, QC, Canada.*
10. *Institute of Systems Neuroscience, Heinrich Heine University Düsseldorf, Düsseldorf, Germany.*

Supplementary results

Asymmetry along each organisation axis

Figure S1A shows the group-mean asymmetry index (AI) maps along the three gradients. Overall, intra-hemispheric AI maps are highly similar to inter-hemispheric AI maps. It describes most leftward (blue) in default-mode and language-related cortices and most right (red) in frontoparietal cortices along G1, most leftward in postcentral cortices and most rightward in posterior temporal cortices along G2, and most leftward in the somatomotor areas and most rightward in medial prefrontal cortices along G3. Cohen's D maps are also shown here to indicate the asymmetric effects in each group as well as the Human Connectome Project (HCP) S1200 template (**Figure S1B**). We performed spatial correlation between individuals with autism/non-autistic individuals (NAI) and HCP in Cohen's maps respectively and showed the Person r values with 95% bootstrap confidence intervals here (**Figure S1B**). In particular, NAI vs autism: intra-hemispheric G1 was 0.698 (0.605, 0.774) vs 0.599 (0.476, 0.703); intra-hemispheric G2 was 0.494 (0.349, 0.608) vs 0.467 (0.343, 0.575); intra-hemispheric G3 was 0.753 (0.684, 0.805) vs 0.653 (0.567, 0.730); inter-hemispheric G1 was 0.516 (0.406, 0.627) vs 0.512 (0.388, 0.634); inter-hemispheric G2 was 0.407 (0.267, 0.537) vs 0.392 (0.262, 0.502); inter-hemispheric G3 was 0.715 (0.627, 0.778) vs 0.612 (0.506, 0.695).

Regarding the comparisons between autism and NAI along each axis, we showed significant parcels ($p < 0.05/3$) for each gradient (**Figure 1B**). We claimed the G1 results in the main text and stated the G2 and G3 results here (**Table S2**). For intra-hemispheric G2, significant parcels are located in area a24 ($t = -2.712$, $p = 0.007$). For inter-hemispheric G2, significant parcels are located in areas including RSC ($t = -2.550$, $p = 0.011$) and STSda ($t = -2.571$, $p = 0.011$). For intra-hemispheric G3, significant parcels are located in areas including VMV1($t = -2.834$, $p = 0.005$). For inter-hemispheric G3, significant parcels are located in areas including 3b ($t = 2.479$, $p = 0.014$), SFL ($t = -2.901$, $p = 0.004$), 3a ($t = 2.478$, $p = 0.014$), 47l ($t = -2.417$, $p = 0.016$), H ($t = 2.981$, $p = 0.003$), and FST ($t = 3.142$, $p = 0.002$). Check the parcellation document (Glasser et al., 2016) to obtain the parcel label information.

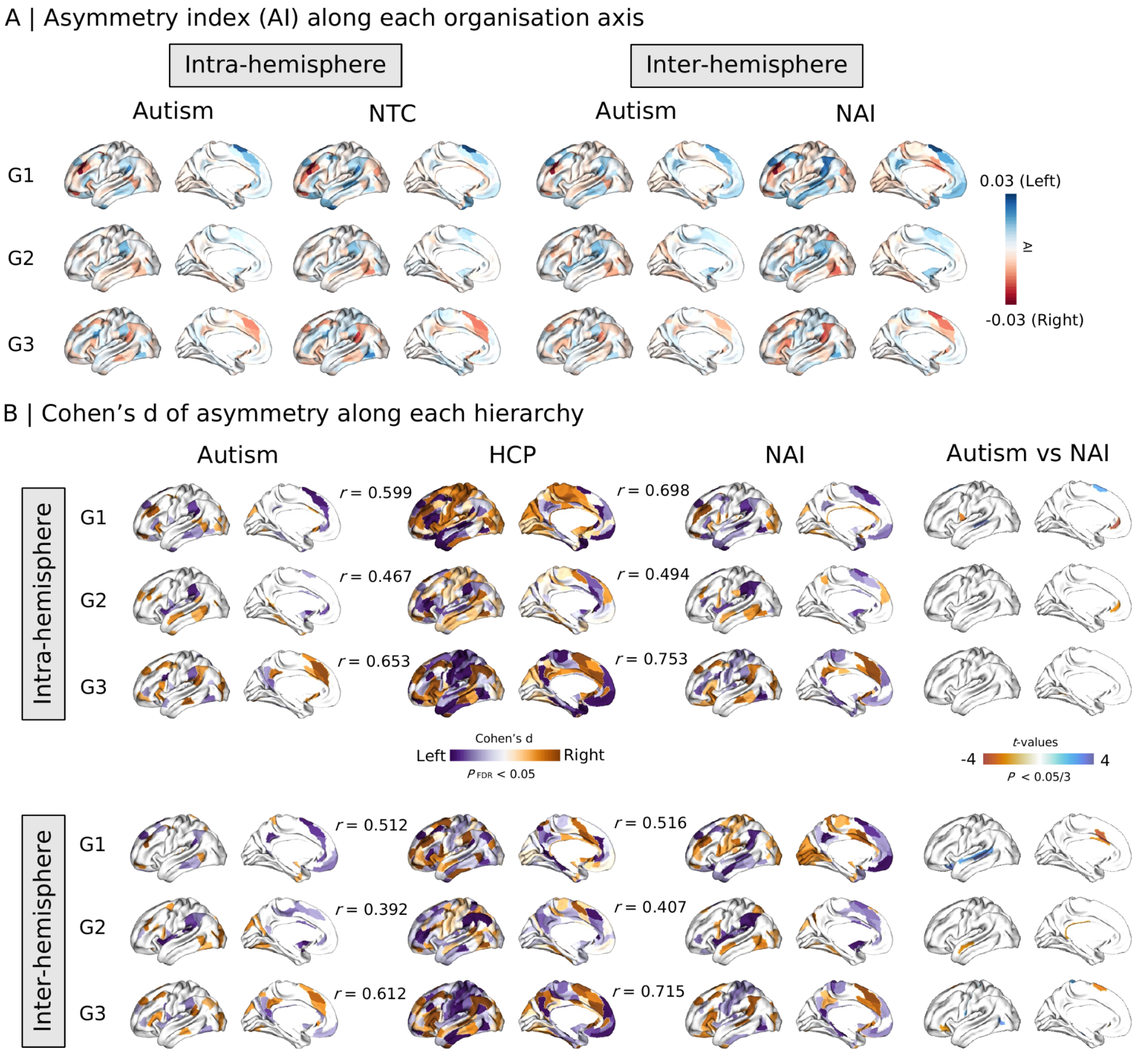

Figure S1. Asymmetry along each axis. **A** | shows the mean asymmetry index (AI) of each group. **B** | shows Cohen's d maps for each group and comparison t-maps between autism and NAI for each axis as well as the spatial correlation between autism/NAI and HCP.

Comparisons between autism and NAI using global signal regression (GSR)

We found the parcels remain significant after GSR and more significant parcels including postcentral cortex and limbic system for intra-hemispheric pattern (**Figure S2**). The network-wise results suggest one more significance in the language network along intra-hemispheric G2. Overall, the main results remain robust after GSR.

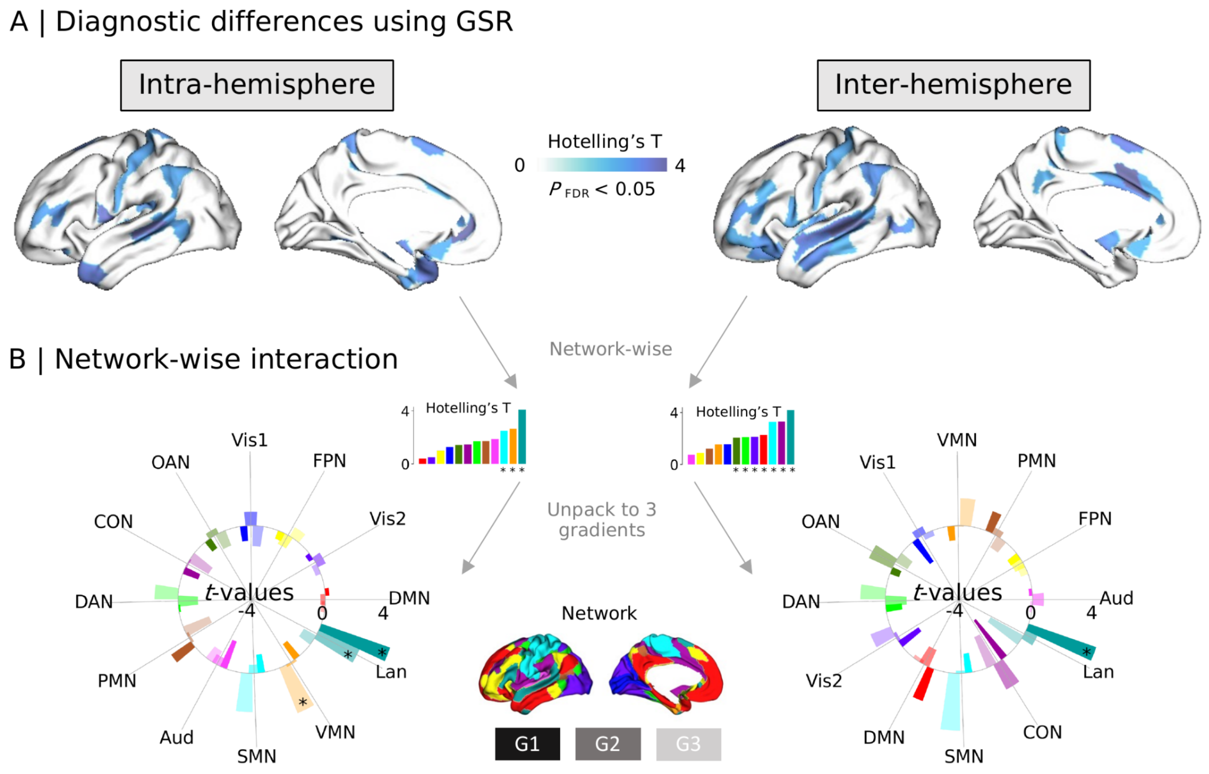

Figure S2. Comparisons between autism and NAI after GSR.

Representational similarity analysis (RSA) across data sites

Critically, the multivariate asymmetric pattern observed between autism and controls may be explained by the idiosyncrasy of autism relative to NAI. Following the previous study (Hahamy et al., 2015) RSA across the 5 data sites was performed to investigate whether the inter-individual AI maps are more heterogeneous in autism relative to NAI in each site specifically (**Figure S3 and Supplementary Table S8**). For the intra-hemispheric pattern, autism showed a lower inter-individual similarity than NAI along G1 (t = -5.119, p < 0.001) driven by Pitt (t = -2.377, p = 0.018), USM (t = -5.331, p < 0.001), and NYU_II (t = -2.312, p = 0.021), and lower similarity along G3 (t = -5.201, p < 0.001) driven by Pitt (t = -3.091, p = 0.002) and USM (t = -6.705, p < 0.001). For the inter-hemispheric pattern, autism showed a lower inter-individual similarity than NAI along G1 (t = -6.540, p < 0.001) driven by NYU_I (t = -2.078, p = 0.038), Pitt (t = -2.827, p = 0.005), and USM (t = -5.292, p < 0.001), lower similarity along G2 (t = -2.561, p = 0.011) driven by Pitt (t = -3.160, p = 0.002) and NYU_II (t = -2.616, p = 0.009), and lower similarity along G3 (t = -7.327, p < 0.001) driven by NYU_I (t = -2.496, p = 0.013), Pitt (t = -3.915, p < 0.001), and USM (t = -7.243, p < 0.001). Overall, this suggests that autism displays more intra-group heterogeneity of cerebral functional asymmetric patterns relative to NAI.

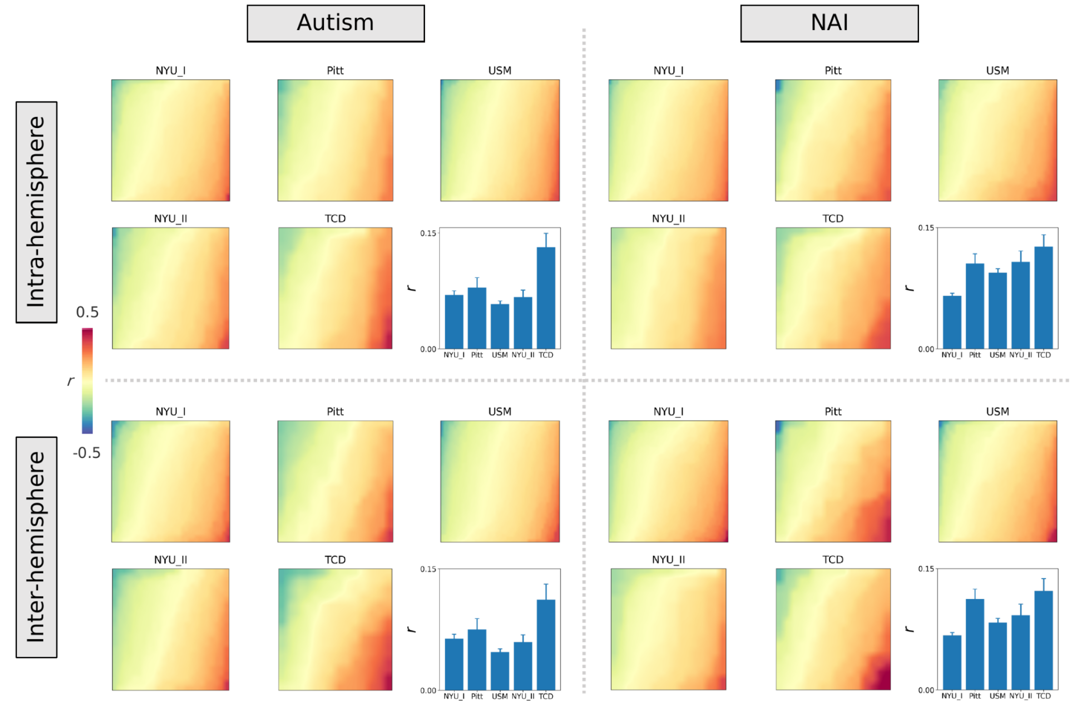

Figure S3. Representational similarity analysis (RSA) across data sites (G1). Inter-subject Pearson correlation was performed to calculate the similarity score. Comparisons between autism and NAI by data sites and gradients were performed. Mean (standard error) values of the similarity of each data site are shown in the bar charts. G2 and G3 results are shown in **Supplementary Table S8.**

Post hoc analyses for the interaction between diagnosis and age

Network-wise interaction results were shown in **Table S5** and their asymmetries by six groups (including NAI children, NAI adolescents, NAI adults, autism children, autism adolescents, and autism adults) in the three-dimensional gradient space were shown in **Figure 2B.** We report specific network-wise *post hoc* results here (**Figure S4**).

- Age differences in autism and NAI (**Table S4**)

Due to three comparisons (children vs adolescents, adolescents vs adults, and children vs adults) here, we set $p < 0.05/3$ (Bonferroni correction) as the significance level. There was no significant age difference in any network. However, in controls, age differences happened in Vis 1 along intra-hemispheric G1 (adolescents vs adults: $t = 3.257$, $p = 0.002$), OAN along intra-hemispheric G1 (adolescents vs adults: $t = -3.090$, $p = 0.003$), Vis 1 along inter-hemispheric G1 (adolescents vs adults: $t = 3.344$, $p = 0.001$), Aud. along inter-hemispheric G2 (adolescents vs adults: $t = -2.451$, $p = 0.016$), OAN along inter-hemispheric G2 (adolescents vs adults: $t = -2.577$, $p = 0.011$), DMN along inter-hemispheric G2 (adolescents vs adults: $t = -2.539$, $p = 0.013$), Lan. along intra-hemispheric G3 (children vs adults: $t = -3.852$, $p < 0.001$), DAN along inter-hemispheric G3 (children vs adults: $t = 2.726$, $p = 0.008$; adolescents vs adults: $t = 2.564$, $p = 0.012$), Lan. along inter-hemispheric G3 (children vs adults: $t = -2.443$, $p = 0.016$), and FPN along inter-hemispheric G3 (children vs adolescents: $t = 2.541$, $p = 0.014$).

- Diagnosis differences in three age groups (**Table S6**)

In particular, we observed the significant diagnosis differences in Lan. along G1 driven by adolescents (intra-hemisphere: $t = -4.182$, $p < 0.001$; inter-hemisphere: $t = -3.843$, $p < 0.001$), Lan. along G3 driven by adults (inter-hemisphere: $t = 2.432$, $p = 0.016$), SMN along G3 driven by adolescents (intra-hemisphere: $t = -2.563$, $p = 0.012$; inter-hemisphere: $t = -2.852$, $p = 0.005$), and VMN along G3 driven by adolescents (intra-hemisphere: $t = -2.798$, $p = 0.006$). While, in the networks having significant interaction, diagnosis differences were observed in Vis1 along G1 in adolescents (intra-hemisphere: $t = 2.485$, $p = 0.015$; inter-hemisphere: $t = 2.594$, $p = 0.011$), and Lan. along G3 in children (intra-hemisphere: $t = -2.993$, $p = 0.004$) and adults (intra-hemisphere: $t = 2.548$, $p = 0.012$).

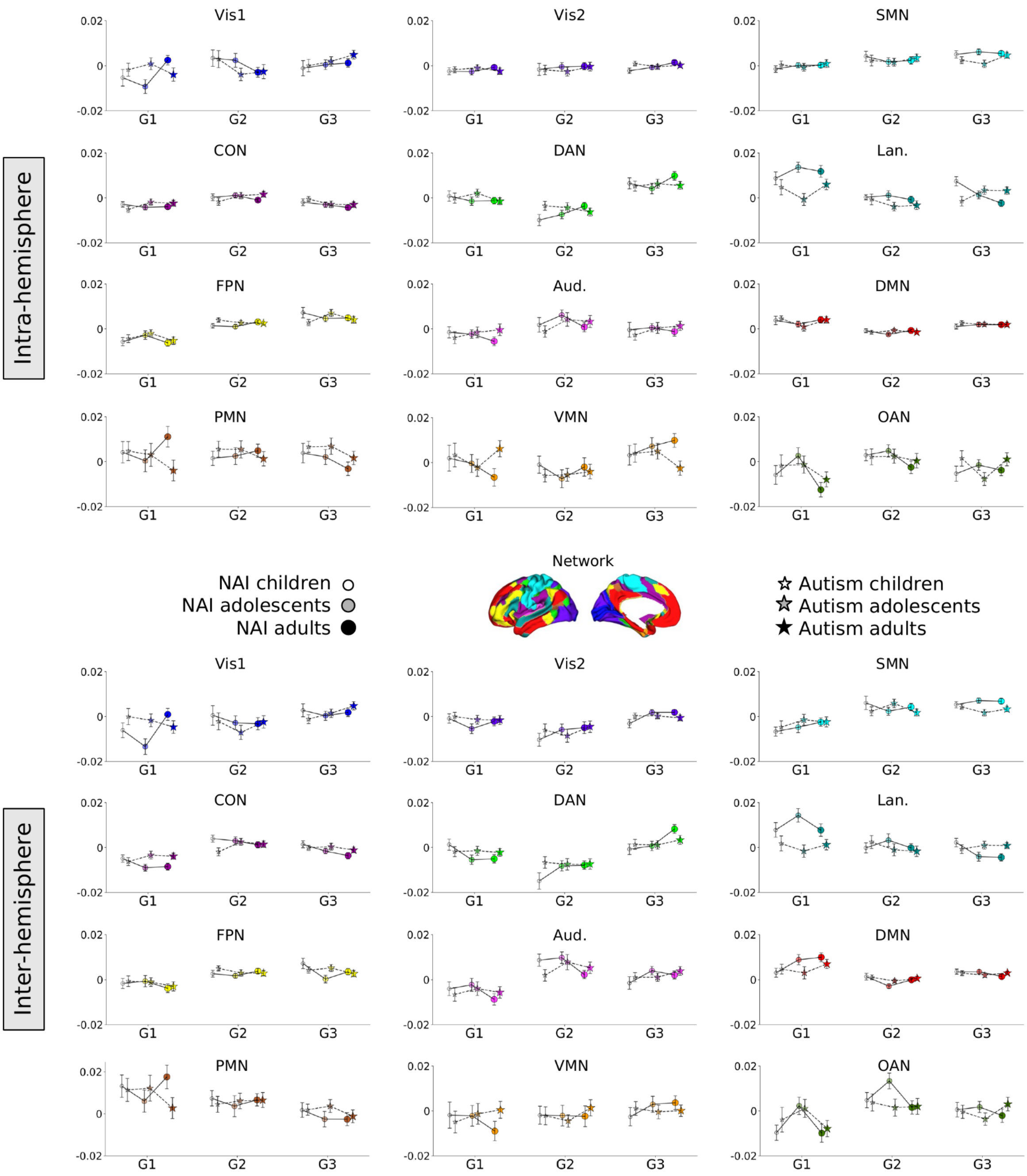

Figure S4. Asymmetry index in age*diagnosis groups by networks.

Interaction between diagnosis and age using GSR

The results remain robust after GSR (**Figure S5**). We still observed significant interaction for intra-hemispheric asymmetry in Lan. along G3 ($t = 3.735$, $p < 0.001$).

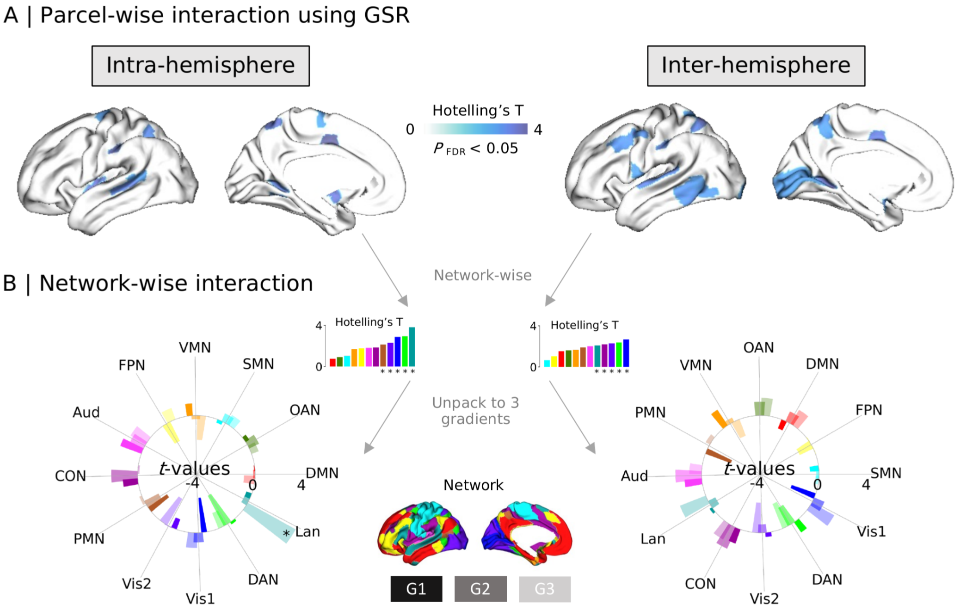

Figure S5. Interaction between diagnosis and age using GSR.

Functional decoding

We decoded the diagnostic comparison maps and interaction of diagnosis and age maps along G1 **(Figure S6)**, G2 **(Figure S7)**, and G3 **(Figure S8)**. The results regarding G1 were also presented in the main text.

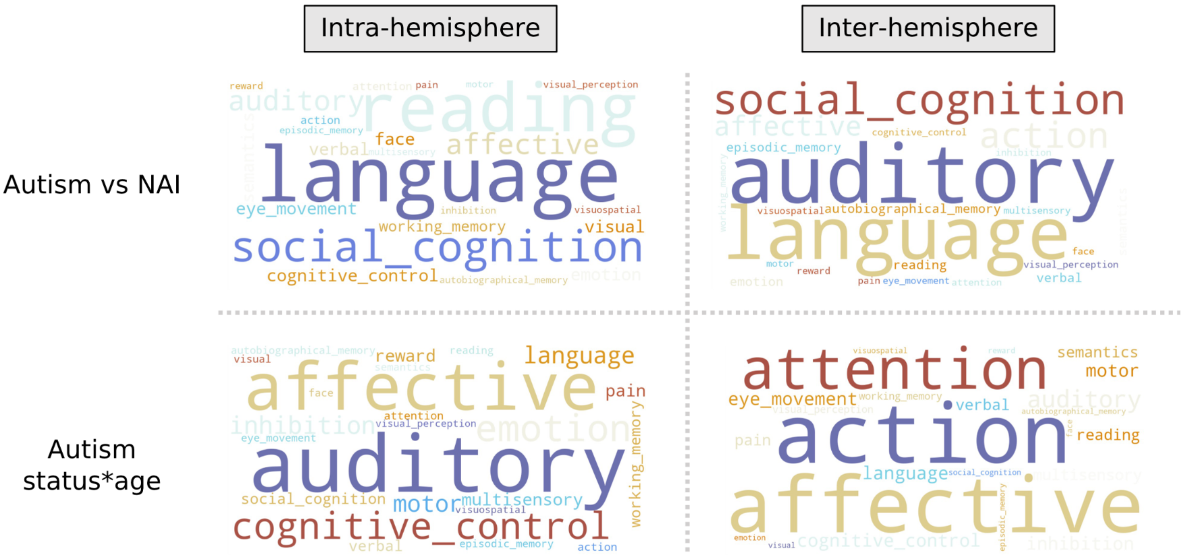

Figure S6. Functional decoding along G1.

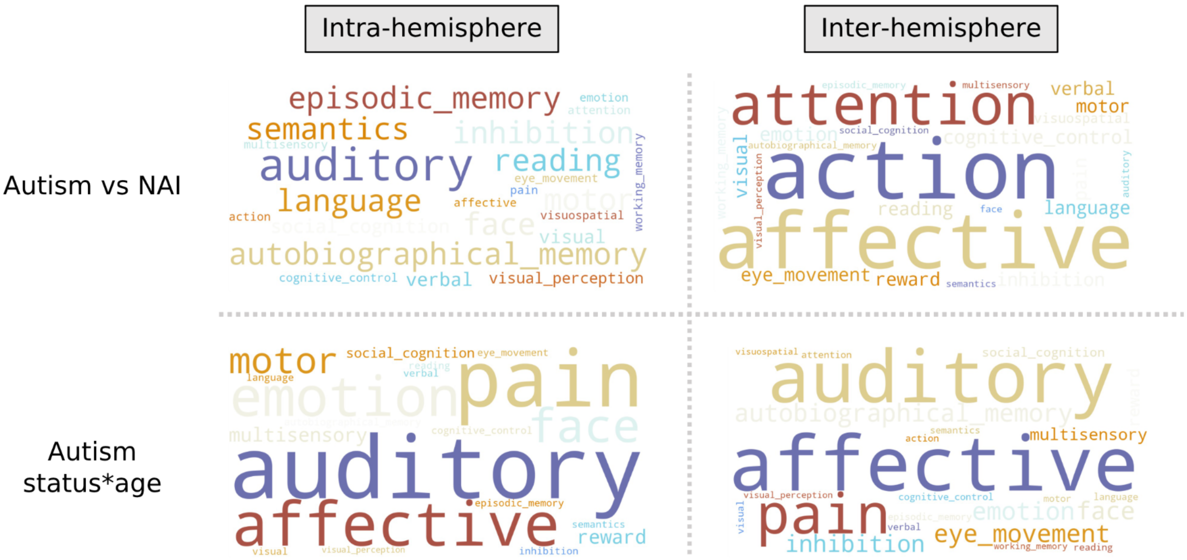

Figure S7. Functional decoding along G2.

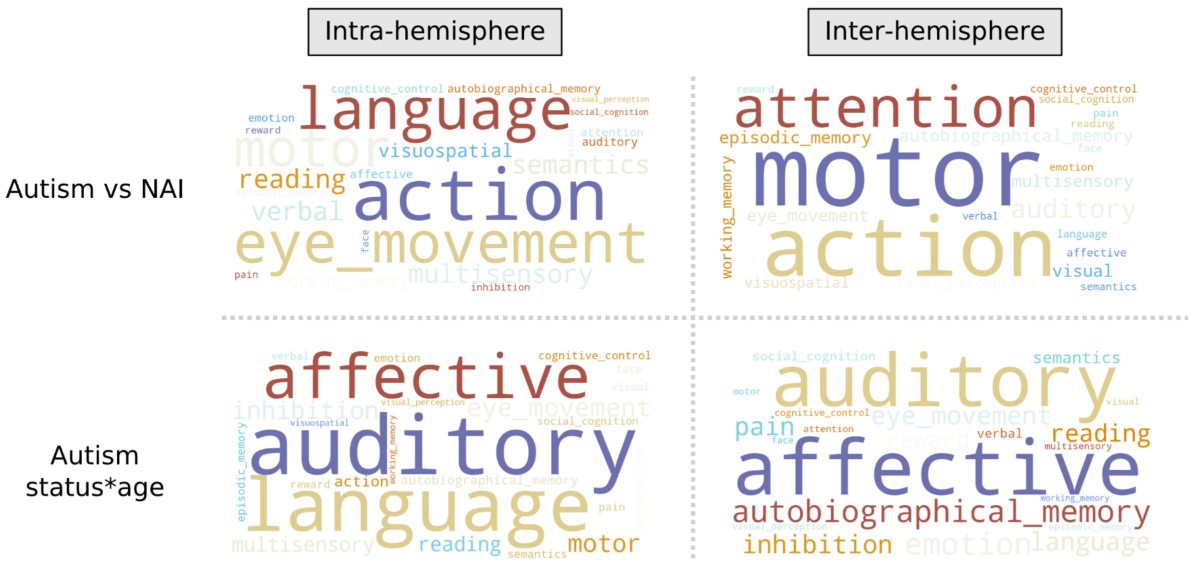

Figure S8. Functional decoding along G3.

Machine learning additional results

1. Choosing L1_ratios

We also performed machine learning using ten L1_ratio models from 0.1 to 1 (**Figure S9**). The performance of the L1_ratio models = 0.1-0.9 worked similarly and the L1_ratio = 1.0 model worked not well using intra-hemispheric asymmetry features. The performance of the L1_ratio = 0.1 model worked best for inter-hemispheric asymmetry features. Hence, we finally chose L1_ratio = 0.1 as the final parameter to set our machine learning pipeline.

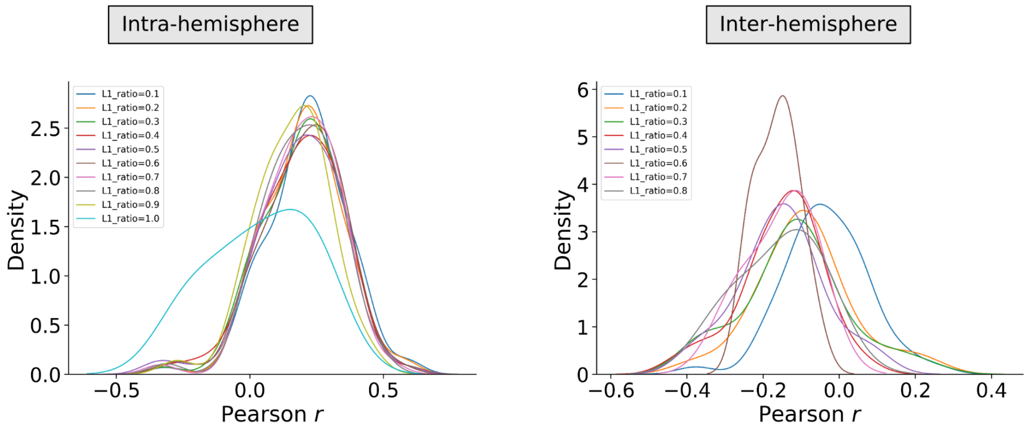

Figure S9. The performance of L1_ratio models = 0.1-1.0 using intra- and inter-hemispheric asymmetry features separately.

2. Predicting ADOS subscores

We used the same pipeline and parameters of cross-validated machine learning to predict ADOS subscores including communication, social, and restricted repetitive behaviours (RRB). In general, intra-hemispheric asymmetry features are working better than inter-hemispheric asymmetry features in predicting communication and social (**Figure S10**).

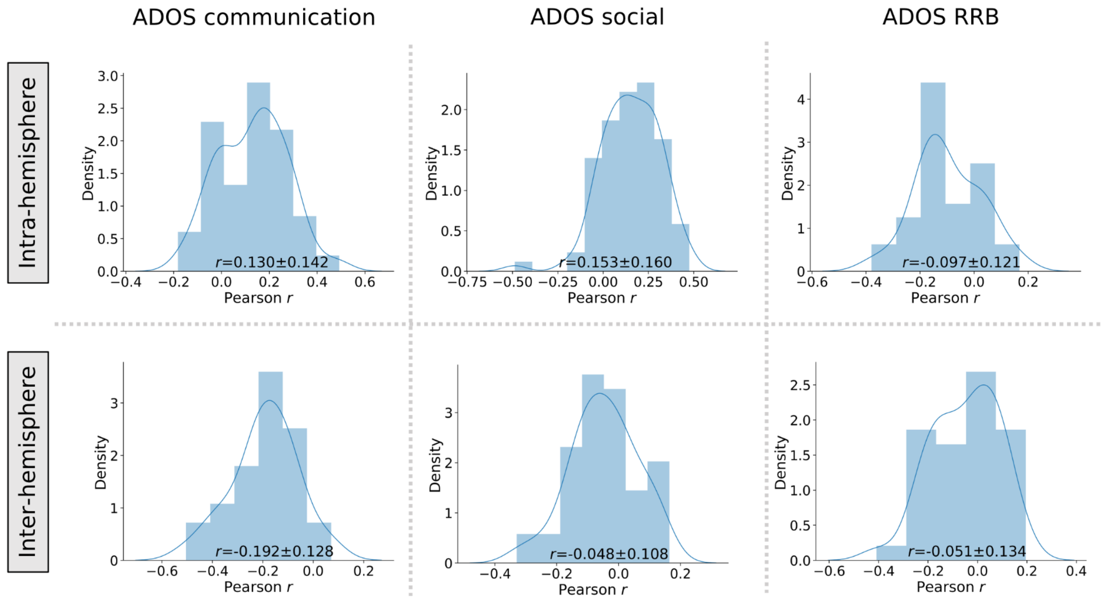

Figure S10. Prediction of ADOS subscores over the 100 permutations.
